# Anti-Inflammatory and Neuroprotective Polyphenols Derived from the European Olive Tree, *Olea europaea* L., in Long COVID and Other Conditions Involving Cognitive Impairment

**DOI:** 10.3390/ijms252011040

**Published:** 2024-10-14

**Authors:** Paraskevi Papadopoulou, Alexia Polissidis, Georgia Kythreoti, Marina Sagnou, Athena Stefanatou, Theoharis C. Theoharides

**Affiliations:** 1 Department of Science and Mathematics, Deree-The American College of Greece, 15342 Athens, Greece; vivipap@acg.edu (P.P.);; 2 Institute of Biosciences and Applications, National Centre for Scientific Research Demokritos, 15310 Athens, Greece; sagnou@bio.demokritos.gr; 3 School of Graduate & Professional Education, Deree–The American College of Greece, 15342 Athens, Greece; 4 Institute for Neuro-Immune Medicine-Clearwater, Clearwater, FL 33759, USA; 5 Department of Immunology, Tufts University School of Medicine, Boston, MA 02111, USA

**Keywords:** neuroinflammation, neurodegenerative diseases, oxidative stress, olive oil, extra-virgin olive oil, blood brain barrier, hydroxytyrosol, oleouropein, oleocathal, phenolic compounds

## Abstract

The European olive tree, *Olea europaea* L., and its polyphenols hold great therapeutic potential to treat neuroinflammation and cognitive impairment. This review examines the evidence for the anti-inflammatory and neuroprotective actions of olive polyphenols and their potential in the treatment of long COVID and neurodegenerative diseases such as Alzheimer’s disease (AD), Parkinson’s disease (PD), and multiple sclerosis (MS). Key findings suggest that olive polyphenols exhibit antioxidant, anti-inflammatory, neuroprotective, and antiviral properties, making them promising candidates for therapeutic intervention, especially when formulated in unique combinations. Recommendations for future research directions include elucidating molecular pathways through mechanistic studies, exploring the therapeutic implications of olive polyphenol supplementation, and conducting clinical trials to assess efficacy and safety. Investigating potential synergistic effects with other agents addressing different targets is suggested for further exploration. The evidence reviewed strengthens the translational value of olive polyphenols in conditions involving cognitive dysfunction and emphasizes the novelty of new formulations.

## 1. Introduction

The ancient Greeks believed that the goddess of wisdom, Athena, created the olive tree. In antiquity, the *Olea europaea* L. tree and its products played an important role as early as the Middle Minoan period, with inscriptions of the words olive and olive oil on Linear A tablets [1,2]. Linear B tablets elucidate the use of olive oil, not only for consumption and cooking but also in the perfume and unguent industry [3]. Theophrastus, in his work “Concerning Odors” (Περὶ οσμών), includes recipes and information about the ingredients used to produce scented olive oil [4]. Greek athletes ritually rubbed it over their bodies before training or competing. The health benefits of olive oil were also known in the ancient world. The *Ebers Papyrus*, a medical text dating back to ancient Egypt, around 1550 BC, contains recipes and remedies for various disorders, many of which include the use of olive oil [5]. Ancient Greeks also believed that olive oil had medicinal properties. Homer called it “liquid gold” and Hippocrates “great healer” [6]. It was recommended as a treatment for skin conditions and digestive disorders [7] and as a form of birth control [8]. Dioscorides, in “*De Materia Medica*” (Περὶ ὕλης ἰατρικῆς) [7], recommends the use of olive oil (green olive oil) for toothaches and headaches.

Many studies have confirmed the medicinal properties of products derived from the olive tree, *Olea europaea* L., and have identified the active compounds for a variety of pharmacological effects. Herein, the anti-inflammatory and neuroprotective properties of olive polyphenols are discussed in relation to brain fog, long COVID, and neurodegeneration.

The Mediterranean Diet (MD)’s growing popularity is due to its healthy and anti-aging properties, particularly in reference to olive oil, as the review by Rigacci et al. (2016) indicates. The review explores the biochemical and physiological correlations of olive tree polyphenols and their derivatives in olive oil, their clinical and epidemiological relevance, and their potential against age-related diseases [9]. Olive oil and the MD are linked to longevity, supported by ecological and epidemiological studies. Biochemical studies and randomized clinical trials show that olive oil’s antioxidant potential and high monounsaturated lipid content are essential for its beneficial effect [10]. Another study on Greek olive oil polyphenols found significant health benefits. Polyphenols like hydroxytyrosol (HT), tyrosol, oleacein, and oleocanthal (OC) were linked to specific parameters like geographical origin, production, and cultivation practice. The study found that OC and oleacein activated healthy aging-promoting pathways and suppressed oxidative stress in mammalian cells and in the Drosophila in vivo model [11].

In this review, we first briefly review the pathological hallmarks of long-term COVID, Alzheimer’s disease (AD), Parkinson’s disease (PD), and multiple sclerosis (MS). Understanding the pathological hallmarks and elucidating the cellular and molecular mechanisms of neurodegenerative diseases, including long COVID, are imperative for the development of effective therapies. Identifying novel therapeutic targets becomes crucial for the development of targeted interventions that extend beyond symptom alleviation to disease prevention and modification. While AD, PD, and MS share common features of protein misfolding, inflammation, and neuronal damage, each disease presents distinct pathological features and clinical manifestations. In contrast, long COVID has so far been associated with disruption of the blood-brain barrier (BBB) and activation of microglia [12]. Thus, further research into the intricate cellular and molecular mechanisms underlying these disorders will pave the way for the development of innovative treatments aimed at improving patient outcomes.

### 1.1. Overview of Neuroinflammation and Brain Fog in Neurodegenerative Diseases and Long COVID

Neuroinflammation and its effects on neurodegenerative diseases pose significant challenges and burdens to public health, with conditions such as AD, PD, and MS affecting millions worldwide [12,13,14,15,16,17,18,19,20,21,22,23,24,25,26,27,28,29]. Recent findings have shown a correlation between COVID-19, cognitive impairment commonly known as “brain fog” [30,31,32,33], and neurodegeneration [34,35] implicating COVID-19 in the future development of neurodegenerative diseases.

The recent COVID-19 pandemic has further highlighted the importance of understanding the neurological complications associated with viral infections, particularly in long COVID patients [12,33,36,37,38,39,40,41,42,43,44,45,46,47]. COVID-19, primarily affecting adults and women, causes severe respiratory problems but may also cause long COVID syndrome, which is characterized by fatigue and cognitive dysfunction, including sleep disturbances, anxiety, depression, attention deficit, and post-traumatic stress, lasting for at least two months and cannot be attributed to an alternative diagnosis [48]. The paper by Theoharides et al. (2021) describes how COVID-19 often causes severe respiratory problems and long COVID syndrome, primarily affecting cognitive dysfunction and fatigue. Symptoms, including brain fog, are similar to those experienced in cancer patients, myalgic encephalomyelitis (ME), chronic fatigue syndrome (CFS), and mast cell activation syndrome (MCAS). The authors propose a phytosomal formulation in olive pomace oil of the natural flavonoid luteolin [32] to mediate those symptoms. Overall, the pathogenesis of brain fog is unknown [32,49,50].

According to the Global Burden of Diseases, Injuries, and Risk Factors Study (GBD) [51] reporting health metrics based on 2021, with a particular emphasis on changes in mortality and life expectancy that occurred, 16 million people died during the 2020–2021 COVID-19 pandemic, representing 12% of all deaths, and life expectancy decreased in 84% of nations and regions. From 1950 to 2021, the average global lifespan rose by nearly 23 years, reaching 71.7 years from 49. However, there was a decrease of 1.6 years between 2019 and 2021 due to the COVID-19 pandemic [51]. The prevalence of long COVID in those affected by SARS-CoV-2 was observed at 77.7%, with the most commonly reported residual symptoms being fatigue (64.1%) and cough (43.9%) [48].

Neurodegenerative disorders are characterized by progressive neuronal dysfunction and degeneration in the central nervous system (CNS), blood-brain-barrier (BBB) dysfunction, increased oxidative stress, and neuroinflammation leading to debilitating cognitive and motor dysfunction, including impairments in visuospatial and executive functions, working memory, abstraction, and orientation [52]. The putative mechanisms of cognitive dysfunction in long COVID include viral persistence, activation of complement and platelet aggregation leading to microthrombosis, fusion of neurons and glial cells, neuroinflammation, impaired neurogenesis, and vagal signaling associated with low serotonin [12,53,54,55,56,57,58,59,60,61].

#### 1.1.1. Alzheimer’s Disease (AD)

AD is the most prevalent neurodegenerative disease, with >55 million cases worldwide, and affects 60–70% of global dementia cases. It is pathologically characterized by the accumulation of amyloid-β (Aβ) plaques and intracellular neurofibrillary tangles (NFTs) known as tau protein tangles in the brain, leading to synaptic dysfunction and neuronal loss and is associated with cognitive decline and memory loss [13,62,63,64,65,66]. Furthermore, AD pathogenesis is associated with dysregulated neurotransmitter signaling, inflammation, and oxidative stress, contributing to about 60–80% of all dementia cases [13]. The current treatment options include acetylcholinesterase inhibitors, n-methyl-d-aspartate (NMDA) antagonists, and monoclonal antibodies targeting Aβ [13], but are generally ineffective.

#### 1.1.2. Parkinson’s Disease (PD)

PD is the most common movement disorder and affects approximately 1% of the global population over the age of 60. It is estimated that almost 10 million people worldwide are living with PD. PD is characterized by the degeneration of dopaminergic neurons in the substantia nigra. Reduced dopamine levels along the nigrostriatal axis lead to motor dysfunction, characterized by four cardinal symptoms: bradykinesia, tremors, postural instability, and rigidity [15,18,24,25]. The key molecular features of PD are α-synuclein (α-syn) aggregation, the main constituent of pathological Lewy bodies within neurons, along with mitochondrial dysfunction and oxidative stress. Drugs such as levodopa (L-DOPA), carbidopa, dopamine agonists, MAO inhibitors, and anticholinergics are commonly prescribed to help manage motor symptoms [15,25]. These medications work by partially replenishing dopamine levels in the brain or by mimicking the effects of dopamine. Physical therapy, occupational therapy, speech therapy, and sometimes deep brain stimulation (DBS) are also used to treat PD patients. While current treatments for PD focus on symptom management, future therapies may offer disease-modifying effects and personalized approaches by targeting underlying disease mechanisms or gene and stem cell therapy approaches [15,18,24,25,67,68].

#### 1.1.3. Multiple Sclerosis (MS)

In MS, infiltration of immune cells into the CNS leads to inflammation, demyelination, and axonal damage [18,67,68]. Dysregulated immune responses, breakdown of the BBB, and activation of microglia and astrocytes contribute to MS pathology. Treatment for MS typically involves a combination of medications that include interferon-beta (IFNβ), such as glatiramer acetate, fingolimod, dimethyl fumarate, and others to either prevent immune cells from entering the brain or inhibit their activities. Acute courses of high-dose corticosteroids, such as oral prednisone or intravenous methylprednisolone, are used to manage symptoms and treat flare-ups, while dietary and lifestyle modifications are aimed at slowing disease progression and improving quality of life. Treatment is often individualized based on the type and severity of symptoms, disease activity, and individual preferences [18].

#### 1.1.4. Coronavirus Disease-2019 (COVID-19)

It is now clear that COVID-19 can affect the CNS, and patients diagnosed with COVID-19 may develop neurological symptoms, including brain fog [30,31,32,33,69]. A retrospective analysis of over 200,000 patients in the UK found that 1.74 and 0.26% of patients admitted to the intensive therapy unit (ITU) due to COVID-19 infections developed dementia and PD, respectively, in the 6 months after initial infection [70]. In attempts to explain this finding, neurodegenerative biomarkers like neurofilament light chain (NfL) and glial fibrillary acidic protein (GFAP) were measured and found to be higher in COVID-19 patients than non-COVID-19 patients with mild cognitive impairment or AD [71]. These markers were correlated with the severity of COVID-19 [34]. Individuals with pre-existing dementia or PD are more susceptible to severe COVID-19 and higher mortality rates. Case reports have documented the emergence of acute PD, AD, or amyotrophic lateral sclerosis (ALS) following COVID-19, with some patients experiencing worsened symptoms after contracting the virus [35,72]. The SARS-CoV-2 virus, responsible for COVID-19, can potentially invade the CNS, leading to neurological symptoms [73,74,75] and causing neuronal damage, primarily due to the transient and persistent stimulation of endothelial cells and microglia by the spike protein located on the membrane of the virus and required for target cell recognition [50]. Recent studies did not detect spike protein in the brains of COVID-19 patients, but they looked only at CSF [76] or in brain regions of very few patients [77]. Evidence from experiments with human brain organoids and autopsies of COVID-19 patients indicates significant SARS-CoV-2-induced neuronal death [78]. The immune responses and cytokine storm triggered by COVID-19 may lead to neuroinflammation and neurodegeneration [35,72].

SARS-CoV-2 Spike enters host cells by binding to its receptor human angiotensin-converting enzyme 2 (hACE2) through its receptor-binding domain (RBD) [73] in some COVID-19 patients [74,79]. Studies have demonstrated that SARS-CoV-2 can infect neural tissues and result in substantial neuronal death, as evidenced by experiments with human brain organoids, mice over-expressing ACE2, and autopsies of COVID-19 patients [74,78,80,81].

Cytokines released during severe COVID-19 infections may cross the BBB, causing direct neurotoxicity and activating microglia and astrocytes [82]. Peripheral immune cells infiltrating the brain may further contribute to neuroinflammatory and neurodegenerative processes. Severe COVID-19 cases have been associated with a cytokine storm featuring increased levels of proinflammatory cytokines like interleukin (IL)-1, IL-6, and tumor necrosis factor (TNF)-α, which may promote neuroinflammation and neurodegeneration [83]. Proinflammatory cytokines may directly induce apoptosis in neurons and breach the BBB, allowing inflammatory cells to enter the brain leading to chronic neuroinflammation and neuronal death. Activation of the NLRP3 inflammasome during SARS-CoV-2 infection could contribute to tau aggregation and neurodegeneration, possibly through the interaction of viral proteins with NLRP3 [84]. Finally, evidence suggests that SARS-CoV-2 infects human monocytes, leading to NLRP3 activation and cell death [84,85].

Clinically, patients with dementia have higher severity and mortality rates with COVID-19. Similarly, individuals with PD experience worsened motor and nonmotor symptoms after contracting COVID-19 [86]. Furthermore, several case reports have documented the onset of acute PD, AD, or ALS following COVID-19 infection [87,88]. Clinical observations suggest that patients with dementia or PD experience higher severity and mortality with COVID-19.

However, observational studies have limitations in establishing causation. Therefore, whether COVID-19 triggers neurodegeneration remains uncertain. It is crucial to acknowledge potential biases in observational studies that link the severity and duration of COVID illness experience with cytokine production, and neuroinflammation might shed light on the process of degeneration [49].

#### 1.1.5. Long COVID

As if the COVID-19 pandemic was not enough, as many as 50 percent of patients infected with SARS-CoV-2 develop post-acute sequelae of SARS-CoV-2 (PASC), commonly referred to as long COVID syndrome, with various symptoms including “brain fog” weeks to months after the initial infection regardless of the severity of the disease [89,90]. Long COVID has been considered as the “Next national health disaster” for the United States (US) [50] and could cost the economy as much as $4 trillion.

Long COVID is characterized by persistent fatigue, various neuropsychiatric, neurological, and neurodegenerative issues, as well as cognitive deficits or impaired consciousness, often described as “brain fog”. The exact mechanism(s) involved in the pathogenesis of Long COVID remains elusive [50,91]. The available evidence indicates that SARS-CoV-2 does not infect brain cells. Instead, the SARS-CoV-2 Spike protein may enter the brain from the nose through the nasal neural mucosa, following the olfactory nerve tract, leading to neuroinflammation that can damage brain blood vessels and neurons. Perivascular inflammation has been reported in the brains of COVID-19 patients, along with evidence of BBB disruption [92]. Autopsy studies of patients with COVID-19 showed severe neuronal loss in the capillaries of the choroid plexus and damage of choroid plexus cell types, as well as neuronal necrosis and glial cell hyperplasia Recent evidence and our studies indicate that the SARS-CoV-2 Spike protein can directly activate the unique immune cells, mast cells and microglia [92], leading to perivascular neuroinflammation, brain endothelial dysfunction, BBB disruption and reduced blood flow to the brain [93]. In particular, we recently reported that recombinant SARS-CoV-2 Spike protein stimulates human mast cells [94] and microglia [32] to release proinflammatory and neurotoxic molecules via activation of different receptors, such as the ACE2 and toll-like receptor 4 (TLR4). Brain fog and various long COVID symptoms, described in various studies, are summarized in Table 1.

Preclinical studies also support a link between SARS-CoV-2 infection and PD- and AD-related neurodegeneration. In hamsters intranasally infected with SARS-CoV-2, neuroinflammation is present in the olfactory bulb, and α-syn and tau accumulate in the cortex, recapitulating the hallmark pathological features of PD and AD, respectively [101,102]. SARS-CoV-2 proteins interact with α-syn and increase its expression in vitro [103]. Furthermore, female animals exhibit a more pronounced response, correlating with changes in myeloid cell density [104]. In infected non-human primates, α-syn accumulations were found in the midbrain several weeks after recovery [105].

### 1.2. Importance of Polyphenols in Addressing Neuroinflammation in Cognitive Health

Consumption of olive oil is associated with reduced overall morbidity and mortality in neurodegenerative diseases [97,106,107,108,109,110,111,112,113,114,115,116,117,118,119,120,121]. Thus, attention has turned to natural compounds with potential anti-inflammatory and neuroprotective properties [121,122,123,124,125,126,127], mainly polyphenols derived from *Olea europaea* L., such as hydroxytyrosol (HT) [128], oleocanthal (OC) [70,129,130] and oleuropein (OL) [123,124,126,127,131]. Polyphenols are naturally occurring compounds present in a wide variety of fruits and vegetables, as well as their derivatives, such as olive oil. The term polyphenols is used to indicate compounds with phenolic moieties (hydroxyl groups attached to benzene rings).

Various preclinical and clinical trials have highlighted the protective properties of olive oil and its phenolic compounds on well-characterized neurodegeneration pathways, linked to multiple putative mechanisms, and, importantly, with no reported toxic effects [109,113,115,132,133,134,135,136].

### 1.3. The Chemistry of Polyphenols in Olea europaea L. Extracts

Olive oil is composed primarily of lipophilic components, rich in monounsaturated fats and a polar fraction of phenolic compounds [137]. These components play an important role in the Mediterranean diet’s health benefits [138]. Alongside its known benefits in lowering lipid and blood glucose levels, olive oil also exhibits anti-inflammatory and antioxidant properties [139]. Olive fruits contain several types of phenols, mainly tyrosol derivates, phenolic acids, flavonols, and flavones [140].

**Figure 1:**
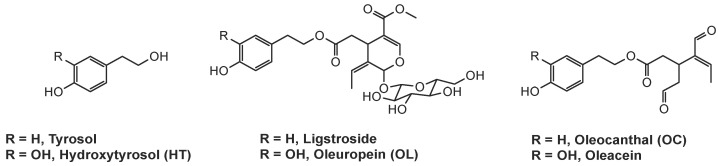
Structures of the biologically active compounds of *Olea europaea*.

The two bioactive secondary metabolites found in olive extracts (Figure 1), phenolic alcohols tyrosol; and HT, in the presence of elenolic acid derivatives, undergo esterification resulting in their glycosylated analogs, ligstroside and OL respectively. It has been proposed that their degradation by specific endogenous esterases [141,142] and β-glucosidases results in the corresponding decarboxymethylated aglycons, OC, and oleacein [143].

The distribution and concentration of phenolic compounds in olive extracts can be significantly affected by a variety of factors ranging from the genetic origin of the plant and the ripening stage to the processing and storage conditions [144,145] that can alter the activity of endogenous enzymes involved in the biosynthesis of these compounds [146,147]. Olive oil chemical profiling, extraction methods, and quality control were reviewed recently [148,149].

Due to these variations, contradictory results can be found in the literature, but most researchers agree that HT, OL, and OC are the main phenolic phytochemicals of therapeutic interest [128,150,151,152,153,154]. Notably, the processing of olive fruits in the production of edible olives and olive oil can affect the composition of several components, such as OL, which is present in small quantities or even absent in the final product, olive oil [117,137,155]. Olive leaves, on the other hand, represent an exploitable, sustainable alternative source of OL, as studies indicate that it is the predominant phenolic compound found in leaf extracts [122,156,157], in addition to HT [114,140,158,159,160].

Extracting the targeted bioactive compounds from natural sources is a critical step toward their isolation and subsequent exploitation; therefore, the employment of suitable extraction methodologies from agricultural leftovers and transformation residues like olive leaves is noteworthy. Traditionally, extraction of phenolic compounds from olive leaves is performed using maceration in organic solvents, resulting in low extraction yields since polyphenols are sensitive to high temperatures [161]. Consequently, new methodologies have been developed in recent years, such as ultrasound, microwave, pressurized liquid, and supercritical fluid properties, to overcome the limitations [162,163,164]. Different assay methods have been used to identify additional bioactive molecules [165,166].

Residual biomasses resulting from agro-industrial processes, such as olive leaves, are, as previously mentioned, rich in bioactive compounds. The extraction and isolation of these compounds as ingredients for a variety of applications in the food, pharmaceutical, and cosmetic industries are therefore desirable for the emerging bioeconomy [149,167,168]. Minor components of olive oil include the flavonoids, luteolin and apigenin [169,170], as well as the luteolin-7-*O*-glucoside [171]. Moreover, luteolin-4′-*O*-rutinoside, luteolin-7,4-*O*-diglucoside, and luteolin-7-*O*-rutinoside were selected from 222 compounds in the oliveNet ^TM^ database as strongly binding to proteins relevant to AD [172]. Luteolin and its derivatives are considered important in the management of many neuroinflammatory conditions [173,174,175,176].

The absorption, distribution, metabolism, and excretion (ADME) properties of olive tree polyphenols (OPs), which are crucial for their nutritional efficacy and toxicological impact, have been partly examined in the review by Galmez et al. (2021). The review provides a comprehensive perspective on ADME processes, potentially aiding future nutritional and toxicological studies [177]. An earlier study by de Bock et al. (2013) investigated the bioavailability and metabolism of phenolic compounds from the olive plant, specifically HT and OL. Nine volunteers were given either encapsulated or liquid OL, and their plasma and urine samples were collected 24 h post-ingestion. The primary metabolites recovered were conjugated HT metabolites. The study found a gender effect on the bioavailability of phenolic compounds, with males showing greater plasma area under the curve for conjugated HT. The study suggests that OL effectively delivers these compounds to plasma in humans [178].

A mouse-based study by Nikou et al. (2024) investigated the metabolic fate of OC in vivo. The results showed that OC was not detected, and 13 metabolites were identified. The study suggests the association of specific metabolites with the biological effects of OC administration, but more research is needed to better understand its metabolism and mechanism of action [179].

### 1.4. Premise and Aims of Study

Olive polyphenols exhibit antiviral, anti-inflammatory, and neuroprotective properties that may alleviate symptoms and improve outcomes in patients with neuroinflammatory and neurodegenerative diseases, including cognitive impairment.

Here, we provide an overview of the chemical nature, properties, and potential therapeutic effects of polyphenols in neuroinflammation, neurodegeneration, and the neurological manifestations observed in long COVID, especially cognitive impairment. We also offer recommendations for further basic and translational research, including clinical applications. HT, OL, OC, and other polyphenols are positioned as promising therapeutic agents that may offer novel approaches to managing these conditions.

Food supplements containing polyphenols are also discussed, including their evaluation and regulation.

An extensive search was performed, spanning the period from 1990 to 2024, focusing mostly on the last five years, on scientific databases including PubMed, Scopus, Google Scholar, and Web of Science in order to identify studies discussing the neuroprotective, anti-inflammatory and antioxidant effects of *Olea europaea* L. polyphenols. The search utilized specific keywords such as “polyphenols”, “hydroxytyrosol”, “oleuropein”, “oleocanthal”, “neuroinflammation”, “neurodegeneration”, “Long COVID”, “COVID-19”, “cognitive decline”, “brain fog” and related terms to locate relevant articles that have been published in peer-reviewed journals. The search was restricted to papers written in the English language. Out of the 450 papers reviewed, 145 were excluded due to their low degree of relevance.

## 2. Results

### 2.1. Neurobiological Effects and Modes of Action of Olive Polyphenols

A substantial body of literature supports the anti-inflammatory, antioxidant, and neuroprotective effects of olive polyphenols in various neuroinflammatory and neurodegenerative conditions [136,180,181,182,183]. Their ability to attenuate neuroinflammation, reduce to oxidative stress, and promote neuronal survival in in vivo and in vitro experimental models is well-documented. The study by Kaddouni et al. (2022) examined the effects of daily consumption of refined olive oil (ROO) and extra-virgin olive oil (EVOO) on brain function and cognitive function in individuals with mild cognitive impairment (MCI) and found that EVOO significantly improved clinical dementia rating and behavioral scores, reduced BBB permeability, and enhanced functional connectivity. The study also found that EVOO biophenols contributed to the effect, suggesting that further clinical trials are needed to assess olive oil’s protective effects against AD and its potential role in preventing MCI conversion to dementias [184]. The review paper by Grubić et al. (2022) provides an updated understanding of olive polyphenols’ beneficial properties and mechanisms of action. The authors state that neurological diseases like stroke and MS are significant medical challenges, and polyphenols from olive trees can alleviate or prevent demyelination, neurodegeneration, cerebrovascular diseases, and stroke. These polyphenols reduce inflammation and oxidative stress, reducing the risk of stroke. They also improve plasma lipid profiles and insulin sensitivity in obese individuals [185].

In addition, according to Infante et al. (2023), olive oil is a key part of the MD, promoting health and preventing chronic diseases. The authors point to the fact that high-quality EVOO is produced in Mediterranean countries and contains the polyphenol OC, among others, which has antioxidant and anti-inflammatory properties. Their review discusses the antioxidant and anti-inflammatory effects of OC, its potential anti-cancer and neuroprotective actions, and the need to include OC content in EVOO nutrition facts labels. The review also discusses the production of certified organic EVOO [186]. Similarly, Boronat et al. (2023) state that olive oil rich in phenolic bioactive compounds has been linked to a lower risk of neurodegenerative diseases and improved cognitive performance in older populations. These compounds can counteract oxidative stress and neuroinflammation, which are linked to age-related cognitive decline. However, the authors stress that there is no direct evidence in humans of the bioactivity of olive oil phenolic compounds. Further research is needed to understand the underlying mechanisms and potential clinical applications [187].

#### 2.1.1. Anti-Inflammatory Properties

HT exhibits potent anti-inflammatory effects by modulating inflammatory cytokines and signaling pathways. In vitro and in vivo, it inhibits the activation of pro-inflammatory transcription factors such as nuclear factor-kappa B (NF-κB) and pro-inflammatory cytokines such as tumor necrosis factor-alpha (TNF-α), interleukin-1 beta (IL-1β), IL-6, and IL-8 [125,188,189,190], which are elevated in patients with severe COVID-19 and contribute to the cytokine storm [32,46,59,94,191,192,193,194]. HT also has been demonstrated to increase levels of the anti-inflammatory cytokine IL-10 [195,196]. In vivo, HT has additionally demonstrated an increase in IL-2, IL-4, and IL-10 and a reduction in IL-17A and TGFβ [123]. By modulating inflammation, HT may alleviate symptoms such as fatigue, muscle pain, and cognitive dysfunction experienced by patients with neurodegenerative diseases or long COVID [81,197].

Like HT, OL also inhibits NF-κB, TNF-α, IL-1β, IL-6, IL-8, and IL-17A [125,188,189,190] and modulates levels of IL-10 and TGF-β [198,199]. OL also decreases the release of TGF-β in LPS-induced RAW246.7 macrophages [177]. In vivo, OL has additionally demonstrated a decrease in IFN-γ and IL-4 [123]. Finally, in a mouse model of Alzheimer’s disease (5xFAD), OL suppresses the activation of NLRP3 inflammasomes and RAGE/HMGB1 pathways [200]. However, one study showed that “nutritionally relevant concentrations” of OL and HT could not inhibit the LPS-stimulated release of pro-inflammatory cytokines from peripheral blood mononucleotides (PBMCs) [125].

OC offers significant potential benefits for neurodegenerative disorders and long COVID [106,132,154]. It can be considered a natural non-steroidal anti-inflammatory drug (NSAID) as it confers dose-dependent inhibition of cyclooxygenase-1 and –2 (COX-1, COX-2), the key enzymes responsible for the synthesis of pro-inflammatory prostaglandins [110]. In fact, at the same dose, OC is a more potent anti-inflammatory agent than ibuprofen. Additionally, it decreases LPS-induced inflammation by reducing expression levels of IL-1β, IL-6, TNF-α, MIP-1^α^, and GM-CSF [70].

The anti-inflammatory properties of HT, OL, and OC’s could mitigate the inflammatory response associated with neurodegenerative diseases and long COVID, potentially attenuating the cytokine storm characteristic of severe COVID-19 cases [32,50,60,191,192,193].

#### 2.1.2. Anti-Oxidant Properties

Oxidative stress, a main contributor to neurodegeneration, is also implicated in the pathogenesis of long COVID and its long-term complications, including tissue damage and organ dysfunction. By neutralizing oxidative stress, olive polyphenols may protect against cellular damage and promote tissue repair in patients with long COVID.

HT acts as a powerful antioxidant by scavenging free radicals and reducing oxidative stress-induced damage to cells and tissues [180,181,201,202,203]. It directly neutralizes reactive oxygen species (ROS) and reactive nitrogen species (RNS), thereby preventing lipid peroxidation, protein oxidation, and DNA damage.

OL, on the other hand, mitigates oxidative stress through activation of antioxidant enzymes such as superoxide dismutase (SOD) and catalase, as well as inhibition of neuronal apoptosis pathways [151,188,204,205]. It reduces inducible nitric oxide synthase (iNOS) activity [198] and activates the Nrf2-ARE pathway, leading to the upregulation of antioxidant genes and the enhancement of cellular antioxidant defenses. Additionally, OL inhibits the activity of enzymes involved in neuroinflammation, such as cyclooxygenase (COX) and lipoxygenase (LOX), thereby attenuating inflammatory responses in the brain.

OC lowers the expression of genes associated with oxidative stress like Nicotinamide Adenine Dinucleotide Phosphate (NADPH) oxidase and enhances the activity of antioxidant enzymes like SOD and Glutathione Peroxidase (GPX) [70,206], and Nicotinamide Adenine Dinucleotide Phosphate Oxidase (NOX) [70].

Overall, these findings highlight the potential therapeutic utility of HT, OL, and OC in neuroinflammatory diseases and neurodegenerative disorders, including those associated with long COVID [144,207,208,209].

#### 2.1.3. Neuroprotective Effects

A number of papers have reviewed the potential neuroprotective actions of olive oil components [186,210]. The molecular mechanisms of action of HT, OL, and OC involve several pathways that could benefit patients with long COVID [127,211,212,213,214].

HT and OL have been shown to inhibit the formation of β-amyloid plaques and tau protein aggregates, thereby mitigating neurodegeneration [70,123,125,126,127,154]. Similarly, in PD, these compounds have been found to protect dopaminergic neurons from oxidative damage and inflammation [106,109,136]. A recent study proposed five olive oil polyphenols as potential nutraceuticals to prevent or reduce the formation of α-syn oligomers that cause PD. The compounds, including vitamin C, were tested in a cellular model and a *Caenorhabditis elegans* PD animal model. Results showed that HT, hydroxytyrosol acetate (HTA), and dihydroxyphenyl acetic acid (DOPAC) effectively inhibited α-syn aggregation in vitro, while dopamine reduced aggregation by 28.7%. DOPAC and HTA were found to be more effective in vivo, demonstrating the potential of olive oil tyrosols as nutraceuticals [215]. Romero-Márquez et al. (2022) also state that HT and OL compounds reduce amyloid-β formation and neurofibrillary tangles. Consumption of olive phytochemicals promotes autophagy and restores proteostasis, reducing toxic protein aggregation in AD models. Thus, according to the authors, olive phytochemicals may be a promising treatment tool [216].

OL shows promise in mitigating neurological complications associated with COVID-19 by targeting proteins involved in neurodegeneration pathways. Molecular docking studies indicate strong binding between OL and target proteins relevant to neurological complications, like TLR-4 and Prolyl Oligopeptidases (POP) [123]. OL protects against neurodegeneration by inhibiting the aggregation of misfolded proteins, such as β-amyloid and α-syn, which are implicated in AD and PD, respectively [200,216,217,218,219]. In addition, it may protect against neurological complications associated with long COVID, such as cognitive impairment and neuropathies. OL inhibits α-syn aggregation and neurodegeneration pathways, which seem to be implicated in long COVID-related neurological symptoms [105,220,221]. OL also enhances BBB integrity and function and improves memory in AD mouse models [200,219]; it also confers neuroprotection in a PD animal model [222]. A recent study reported that consuming 7 g of olive oil daily decreases dementia-related death risk by 28% [223]. By reducing misfolded and aggregated protein burden and preserving neuronal function and integrity, OL may improve cognitive function and alleviate neurological symptoms in patients with long COVID-19 [65,224,225,226]. In neurodegenerative diseases, OC helps by reducing inflammation, clearing amyloid plaques, and protecting against tau pathology [64,106,109,129]. In long COVID, OC’s ability to reduce chronic inflammation, protect neurons, enhance antioxidant defenses, and improve vascular health makes it a promising compound for alleviating persistent symptoms and promoting recovery [227]. Overall, HT, OL, and OC exert their neuroprotective effects through a combination of antioxidant, anti-inflammatory and anti-apoptotic mechanisms, making them promising candidates for the treatment of neurodegenerative diseases and long COVID.

### 2.2. Implications for Long COVID

HT exhibits potential antiviral activity against influenza A virus (IAV), human immunodeficiency virus (HIV), and coronaviruses, including SARS-CoV-2. This compound demonstrates high binding energies to viral proteins, suggesting efficacy in reducing viral virulence. HT has been shown to possess antiviral properties by inhibiting viral replication and attachment. It may interfere with viral entry into host cells by blocking viral receptors or fusion proteins, thus preventing viral infection and spread [227,228,229]. In patients with long COVID, HT’s antiviral activity could help reduce viral persistence and prevent reactivation of the virus, potentially alleviating symptoms and preventing disease progression [32,60,227,228,229].

The recent work by Crudele and colleagues [230] provides solid evidence in vitro that SARS-CoV-2 remaining in host cells after viral clearance may contribute to the pathogenetic mechanisms of long COVID by inducing a cascade of interferon-related inflammatory genes and proteins and increasing the apoptotic rate and expression of several oxidative stress markers in epithelial cells. Treatment with HT restored the expression of pro-inflammatory genes/proteins at levels similar to controls, reduced apoptotic rate and pro-oxidant state, suggesting the potential therapeutic potential of HT against Long-COVID pathologies.

OL exhibits antiviral activity by targeting viral proteins involved in replication and virulence. It may inhibit viral proteases, such as the main protease (3CLpro), essential for viral replication. By blocking viral proteases, OL can disrupt viral replication and reduce viral load, potentially preventing viral persistence in patients with long COVID.

### 2.3. Integration of the Molecular Mechanisms of Action

In addition to the well-known actions of polyphenols discussed above, certain minor components of *Olea europea* L. have been reported to have unique properties beneficial to COVID-19 and Long COVID [231,232]. Prominent among them is the flavonoid luteolin (tetramethohydroxyflavone) [174], which has potent antiallergic and anti-inflammatory actions, is neuroprotective, [175] reduces cognitive dysfunction, especially brain fog [233], and may be used against brain-related disorders [176,234]. Luteolin was reported to inhibit SARS-CoV-2 by binding to ACE2 [235,236], and a luteolin-rich fraction inhibited SARS-CoV-2 Spike protein-induced NLRP3-dependent lung inflammation [237]. Moreover, luteolin 7-*O*-*b*-D-glucopyranoside was identified as an inhibitor of SARS-CoV-2 RNA-dependent RNA polymerase [238], and the luteolin structural analog eriodictyol (tetramethoxyflavanone) was identified as a potent inhibitor of SARS-CoV-2 [239]. Other polyphenolic compounds present in olive oil [122] and olive leaves [227,240], such as OL and HT, have also been found to be powerful SARS-CoV-2 antiviral [241] and serine protease inhibitors [242]. In fact, OL was identified as a potent compound against neurological complications associated with COVID-19 [123]. In addition, berberine has been shown to inhibit the serine protease involved in SARS-CoV-2 entering the target cells [243], and sulforaphane has been reported to inhibit SARS-CoV-2-induced cytokine storms [244]. As discussed earlier, neurovascular inflammation has been identified as a key pathologic event in many neurodegenerative diseases, especially long COVID [12,92]. Platelet-activating factor (PAF) has been implicated in micro-clotting in COVID-19 [228,245,246], and it is interesting that olive components can modulate the activity of PAF [247,248,249,250]. A summary of the key neurobeneficial effects discovered in the last three years is shown in Table 2.

### 2.4. Olive Polyphenols as Dietary Supplements with Potential Clinical Applications

Several in vivo and clinical studies have investigated the effects of supplementing food with olive polyphenols, with promising results, both in terms of neuroprotection and limiting adverse effects [152,251]. Interestingly, even though the use of HT as a novel food ingredient was approved the European Union (Regulation (EC) No 258/97) [251] and the United States [252] for the (GRN876 [US FDA, 2020]; [253] for GRN 600 [US FDA, 2015]), there is to date only a limited number of studies focusing on commercial dietary supplements containing olive extracts or olive polyphenols.

The addition of polyphenols to foods has been reported to improve their nutritional value [156,254]. The EFSA reference cited in the article is a decision where the panel concluded that synthetic hydroxytyrosol is safe to be added to fish and vegetable oils and to margarine (up to 215 mg/kg and up to 175 mg/kg, respectively) for consumption by the general population, excluding children under 36 months of age, pregnant women and breastfeeding women. Nevertheless, our understanding is that aqueous extracts of olive fruit containing more than 10% hydroxytyrosol are considered by the EU as novel foods.

Olife^®^, a food supplement based on an infusion of olive leaves and marigold aqueous extracts (80–95% *Olea europaea* L. extract; [255], and Bonolive^®^, an olive leaf extract supplement, containing 40% oleuropein [256], were included in clinical studies on glyco-metabolic parameters and joint functional capacity, respectively.

An olive juice extract rich in HT, Hidrox^®^, is produced on a large scale from the aqueous effluent of the olive oil industry (olive vegetation water or olive juice), which carries almost 50% of the weight of the olive fruit and is usually discarded as wastewater. The process relies on citric acid (1%) assisted mild hydrolysis of naturally occurring hydroxytyrosol esters, producing HT in high yields [257]. Various studies have demonstrated the beneficial effects of Hidrox^®^ on health in both in vitro and in vivo systems for the regulation of inflammation, neurodegeneration, and Parkinson’s disease [258,259,260]. Recently, Hidrox^®^ solution has been reported to exhibit time- and concentration-dependent SARS-CoV-2-inactivating, virucidal activity. From a mechanistic point of view, Hidrox^®^ was shown to induce structural changes in SARS-CoV-2, which changed the molecular weight of the spike proteins, regardless of their glycosylation status, while also disrupting the viral genome [261].

### 2.5. Fatigue

The chronic effects of supplementation with a biodynamic and organic olive fruit water phytocomplex (OliPhenolia^®^ [OliP]), rich in HT, on submaximal and exhaustive exercise performance and respiratory markers of recovery were investigated by Roberts and colleagues [100]. Twenty-nine recreationally active participants (42 ± 2 yrs; 71.1 ± 2.1 kg; 1.76 ± 0.02 m) consumed 2 × 28 mL∙d^−1^ of OliP or a taste- and appearance-matched placebo (PL) over 16 consecutive days. Consumption of the phytocomplex resulted in increased time to exhaustion and provided some benefits for aerobic conditions and acute recovery.

Another study demonstrated that exercise-induced fatigue and damage to muscle and immune functions are mediated via the regulation of mitochondrial dynamic remodeling [262]. The reversal of the downregulation of mitochondrial biogenesis and upregulation of autophagy by HT supplementation was, thus, accompanied by improvement in endurance capacity and muscle atrophy.

Supplementation with 1200 mg olive leaf extract enriched in OL, containing 16–24% oleuropein and ≥30% olive phenols [263], as nontransparent capsules, for one week in a randomized, balanced, double-blind manner was found to alter the serum and urine metabolomes of athletes compared to the placebo administration. Overall, the findings support the notion that OL administration alters an array of factors concerning crucial biochemical pathways that are implicated in physical condition, feelings of fatigue and muscle pain, and activity readiness, including the upregulation of tryptophan and the increase in the circulating acylcarnitines in the serum and urine [264].

The OL antioxidant effect was found to be an effective agent for olive leaf extract’s effects on obesity, cognitive decline, depression, and endurance exercise capacity in a mouse model [265]. In physically inactive mice fed a high-fat diet, olive leaf extract administration inhibited body weight increases and did not allow the onset of cognitive declines, and more specifically, improved working memory and reversed depressive behaviors. Additionally, olive leaf extract increased endurance exercise capacity under atmospheric and hypoxic conditions.

The study by Rodríguez-Pérez et al. (2022) investigated the neuroprotective effect of 3′,4′-dihydroxyphenylglycol (DHPG) from EVOO in a diabetic model. The effect was evaluated in brain slices and retinal nerve cells. Diabetic rats showed higher levels of oxidative stress and reduced neuronal cell numbers. DHPG or HT administration reduces oxidative stress and brain lactate dehydrogenase efflux, reducing cell death. The combination of DHPG and HT seemed to have improved their neuroprotective and antioxidant effects [266].

In one study of rodent mice, the addition of isoflavones to the chow decreased fatigue and associated blood-brain inflammatory markers [267].

### 2.6. Psychiatric Symptoms

As the COVID-19 crisis developed, the psychological impact of COVID-19-related quarantine has been reported to include post-traumatic stress disorder (PTSD), confusion and frustration [268]. Mental distress, grief and bereavement, deliberate or unintentional harm to family, loss/separation from family, self-injury, shame, guilt, helplessness, addiction or substance use, medical mistrust and inclination towards conspiracies, panic attacks, stress, anxiety, depression, loneliness, suicidal ideation, mood problems, sleep problems, worry, denial, boredom, ambivalence, uncertainty, frustration, anger, fear, stigmatization, marginalization, xenophobia, mass hysteria, socioeconomic status, and other mental health concerns have also been indicated worldwide [269].

The presentation of depression during COVID-19 differs in older adults compared to younger ones [270]. For example, older adults with cardiovascular disease (CVD) and depression are less likely to manifest affective symptoms and are more likely to display cognitive changes, somatic symptoms, loss of interest along with dysfunctional defense mechanisms regarding health problems (e.g., refusal to accept actual state of health) and failure to conform to doctor’s instructions than are younger adults [271,272,273,274,275]. Quarantine measures in the general population have raised a number of issues for mental health. In a study in Australia, the COVID-19 impact and quarantine measures include an array of mental health concerns that may aggravate or trigger existing distress [276]. Rates of elevated psychological distress were higher than expected, with 62%, 50%, and 64% of respondents reporting elevated depression, anxiety, and stress levels, respectively, and one in four reporting elevated health anxiety. Participants with a self-reported history of a mental health diagnosis had significantly higher distress, health anxiety, and COVID-19 fears than those without a prior mental health diagnosis. Higher engagement in hygiene behaviors was associated with higher stress and anxiety levels.

HT may exert an antidepressant activity through its ability to stimulate hippocampal neurogenesis and neuron survival in young and aged mice [277]. Chronic unpredictable mild stress (CUMS) mice are considered one of the most widely accepted mouse models of depression. HT supplementation of such an experimental model has been shown to exhibit a strong anti-depressant effect. More specifically, its anti-depressant activity was associated with reduction of oxidative stress and with increased number of glial fibrillary acidic protein (GFAP)-immunoreactive astrocytes, as well as increased activity of the BDNF/TrkB/CREB signaling pathway [278]. The latter is also involved in stimulating neurogenesis, which is, therefore, the mode of anti-depressant activity of HT revealed by this study. Furthermore, Fan et al. reported [279] that HT exerts a significant antidepressant effect in association with the improvement of the HPA axis. This is implied by the decrease in serum corticosterone, adrenocorticotropic hormone (ACTH), and also TNF-α, IL-1β, and IFN-γ in CUMS mice. Additionally, it was also shown to be capable of reverting the alteration in gut microbiota composition, but only partially, which may imply that this is not the primary anti-depressant mode of action for HT.

In the case of OL, a different anti-anxiety mechanism was proposed involving the ability to restore the levels of hippocampal Neuropeptide Y, which modulates serotonergic pathways, as well as the levels of brain-derived neurotrophic factor (BDNF), as reported by Lee et al. [280]. Additionally, the depression-like symptoms of mice were elicited by OL administration (8 to 32 mg/kg i.p.). This activity was suggested to be caused by restoring the brain’s serotonin and dopamine levels [281].

Using an AD model, 5xFAD mice treatment with OC (10 mg/kg) resulted in improving several of the assessed parameters to levels similar to or approaching those of the wild-type (WT) mice, including sleeping time during the day and anxiety-like behavior [282].

### 2.7. Cognitive Impairment

There is increasing evidence that cognitive difficulties and memory problems are present in the post-acute phase of SARS-CoV-2 infection, which is very frequently compared with and associated with an AD-type cognitive impairment [283,284,285]. Indeed, several reports demonstrate various neuropathological similarities of PASC Cognitive Syndrome with AD, including numerous elevated AD marker genes, including FERMT2, HLA-DRB1, GNA15, STAB1, ICA1L, COLGALT1, TNFAIP2, ITGAM, VASP, IDLIA, PVR, TECPR1, several circulatory biomarkers, such as GFAP, NFL, P-tau 181, UCH, NSE, and S100B, and the presence of Apolipoprotein E4 allele (APOE4) [283,285]. It would, therefore, be reasonable to suggest that any potential anti-AD activity of HT, OL, and OC would be supporting evidence for the high value of these molecules in fighting against long COVID symptoms.

In a *C. elegans* model of AD, the effects of an olive fruit extract 20% rich in HT on the molecular mechanisms associated with AD features like Aβ- and tau-induced toxicity were evaluated. The extract showed a reduction of proteotoxicity associated with the aggregation of the tau protein, whereas, from the RNAi tests, the SKN-1/NRF2 transcription factor and of HSP-16.2 also limited the expression of [286].

In a mouse model study, supplementation with HT significantly improved the cognitive functions of TgCRND8 mice and also reduced Aβ42 and pE3-Aβ plaque area and number in the cortex. In the hippocampal areas of HT-fed TgCRND8 mice, the pE3-Aβ plaque number was also significantly reduced together with a tendency toward a reduction in Aβ42 load, associated with a marked reduction of TNF-α expression and astrocyte reaction. The beneficial effects of HT were attributed to macro-autophagy induction and modulation of MAPKs [287]. Similarly, in APP/PS1 transgenic mice orally treated with HT acetate, improved cognition was witnessed by the escape latency, escape distance, and the number of platform crossings of AD mice in the water maze test by ameliorating neuronal apoptosis and decreasing inflammatory cytokine levels. It was further demonstrated that HT acetate stimulated the transcription of ERβ and enhanced neuronal viability and electrophysiological activity in primary neurons but that these beneficial effects were abolished upon ERβ deficiency [288].

HT was also studied against the learning and memory decline of obese mice. Both abilities were significantly improved, and the expressions of brain-derived neurotrophic factors (BDNFs) and postsynaptic density proteins were enhanced, protecting neuronal and synaptic functions in obese mice. Transcriptomic results further confirmed that HT improved cognitive impairment by regulating gene expression in neural system development and synaptic function-related pathways [289].

Cognitive impairment could be addressed in different ways, but the results are inconclusive [290,291]. The dietary supplement BrainGain^®^ contains a combination of HT, luteolin, calcium folinate, and berberine in olive pomace oil, which increases oral absorption and provides additional polyphenols [9]. Unique aspects of these dietary supplements are that they are made in a GMP-certified facility that is registered with the US Food and Drug Administration (FDA), but that they also have an FDA-issued Certificate of Free Sale renewable every two years. Unfortunately, most other dietary supplements contain individual components of questionable purity and do not comply with regulatory requirements [292].

## 3. Discussion—Future Directions and Challenges

Recently, Filardo et al. (2024) stressed health challenges that are becoming increasingly global, with chronic diseases like cardiovascular, neurological, and respiratory diseases, cancer, and diabetes being major threats along with antimicrobial resistance, which is a growing public health concern. In their review, natural products like olive tree leaves, fruits, and oil are being investigated for their health-promoting properties. *Olea europaea* L secoiridoids, including OL, OC, oleacein, and ligstroside, are promoted for their anti-inflammatory, antioxidant, cardioprotective, neuroprotective, and anticancer activities [116]. Platelet Activating Factor (PAF) is a potent inflammation mediator, contributing to chronic diseases like cardiovascular, metabolic, inflammatory, renal, and neuropsychiatric diseases [60]. The effect of MD on PAF was covered in a systematic review [247]. The protective effect of olive oil microconstituents in atherosclerosis and the role of PAF has been stressed by Antonopoulou et al. [250]. A systematic review of epidemiologic and intervention studies found that healthy MD components, such as cereals, legumes, vegetables, fish, and wine, can modulate PAF’s pro-inflammatory actions. A healthy diet with PAF inhibitors may target inflammation and microthrombosis [245]. Detopoulou et al. (2021) suggested that yogurt enriched with PAF inhibitors could potentially modulate PAF biosynthetic and catabolic pathways [249]. Vlachogianni et al. (2015) examined PAF biosynthesis and showed that it is inhibited by phenolic compounds in U-937 cells under inflammatory conditions [248].

As presented herein, the olive tree produces key bioactive compounds with neuroprotective properties that can be proven effective in the management of conditions affected by neuroinflammation and cognitive decline. In the absence of any published benefit of repurposed drugs, investigating the use of high-quality dietary supplements for long COVID is a prudent next step, as they are safe and potentially quite effective [254,276,293,294,295].

Environmentally and economically sustainable procedures could be applied to olive oil processing on an industrial scale [296], yielding high-purity isolates [297] with neuroprotective benefits, as evident by this review and other clinical studies [13,298,299]. The use of Machine learning and NMR in the detection and quantification of phenolic compounds is also promising [170,300].

Given the findings discussed above, it appears that all complex disorders reviewed involve neurovascular inflammation that may benefit from the introduction of *Olea europaea* L. polyphenols. However, the best approach would require simultaneously addressing various target points. These may include the BBB, entry of leukocytes in the brain, microglia, misfolded proteins, specific receptors, inflammasome, extracellular matrix, generation of inflammatory molecules, matric degrading enzymes, production of neurotrophic factors, etc.

**Figure 2:**
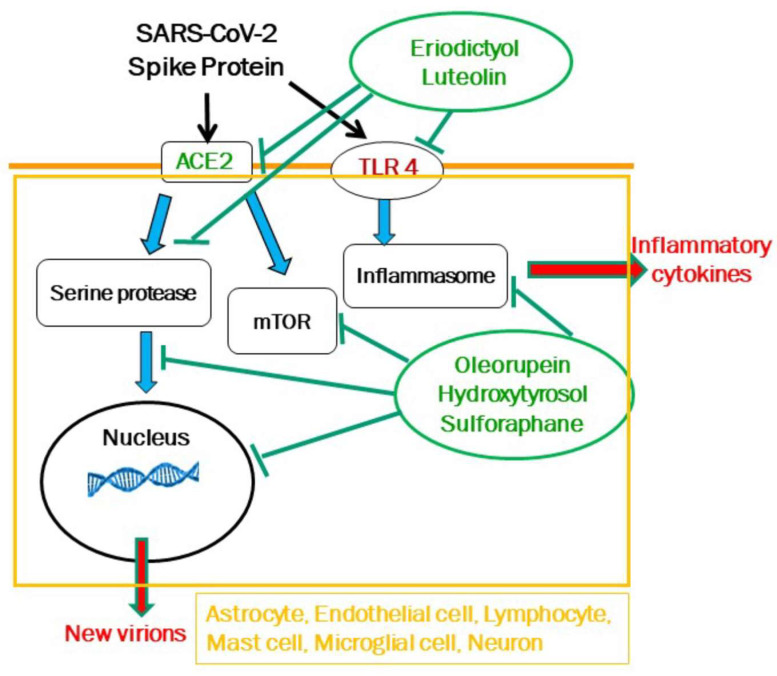
Diagrammatic representation of one generic cell showing how different natural molecules could inhibit multiple targets of SARS-CoV-2. Luteolin and eriodictyol would inhibit both ACE2 and TLR4, while oleuropein, hydroxytyrosol, and sulforaphane would inhibit the serine esterases and RNA polymerases, as well as the mammalian target of rapamycin (mTOR) and the inflammasome. Black arrows indicate activation, green T lines indicate inhibition, blue arrows indicate outcomes, and red arrows indicate secretion extracellularly.

In the case of long COVID, this approach could entail targeting ACE2 necessary for viral binding, serine protases required for viral entry, RNA polymerases for viral multiplication, and TLRs for inflammatory molecule production (Figure 2). For instance, luteolin and eriodictyol, an in silico inhibitor of human ACE_2_ receptor required for SARS-CoV-2 binding to host cells [301], would inhibit both ACE2 and TLR4 [302], while OL, HT, and sulforaphane would inhibit the serine esterases and RNA polymerases (Figure 2). Luteolin has anti-inflammatory properties [173,174,175,176] but is difficult to dissolve in aqueous media and is poorly absorbed in powder form (less than 10%) after oral administration, while eriodictyol offers the advantage of being partially soluble in water. The unique combination of eriodictyol, HT, OL, and sulforaphane is found in the dietary supplement ViralProtek^®^.

Additional mechanistic studies will further elucidate the mode of action and explore the potential synergistic effects of HT, OL, and OC in combination with other related compounds that could improve their beneficial properties. A major impediment to the conduction of mechanistic studies is the lack of relevant in vivo or in vitro models for these diseases. The recent development of human microfluidic organoid brain-on-a-chip models could be used as a disease “surrogate” and would greatly enhance our understanding of pathogenetic mechanisms [303,304,305], such as a system presently used in our laboratory (TCT) for the study of ALS, long COVID, and PD where we also investigate the effect of *Olea europaea* L. polyphenols.

To date, only a small number of marketed food supplements contain combinations of *Olea europaea* L. polyphenols alone or with other relevant natural molecules. Exploring, therefore, novel formulations appears to be a safe and potentially effective way of managing complex neuroinflammatory disorders. Additional clinical trials are also warranted to evaluate the efficacy, safety levels, and long-term effects of these compounds in relation to their neuroprotective properties and their use as targeted food supplements. Unfortunately, there is a lack of interest in funding clinical studies using dietary supplements because they are not covered by patents; instead, interest has focused on repurposing approved drugs, even though such studies have not yielded any significant findings.

## 4. Materials and Methods

In order to conduct this review, an extensive search was performed on scientific databases, including PubMed, Scopus, Google Scholar, and Web of Science. The purpose of this search was to identify studies that have investigated the neuroprotective, anti-inflammatory, and antioxidant effects of *Olea europaea* L. polyphenols, HT, OL, and OC. The search utilized specific keywords such as “polyphenols”, “hydroxytyrosol”, “oleuropein”, “oleocanthal”, “neuroinflammation”, “neurodegeneration”, “Long COVID”, “COVID-19”, “cognitive decline”, “brain fog” and related terms to locate relevant articles that have been published in peer-reviewed journals. Items searched include mostly journal articles, conference papers, and technical reports and reviews. The introduction of the review provides an explanation of the review’s purpose in relation to the existing knowledge. The background section introduces the topic. The study clearly outlines its objectives and research inquiries. The eligibility criteria for both the inclusion and exclusion criteria were determined, and the studies were categorized based on their primary focus. Inclusion criteria were determined based on the pertinence to the subject matter and the quality of the evidence. Data collection involved extracting information regarding the study’s design, participants, interventions, outcomes, and significant conclusions and discoveries.

The comprehensive analysis pertains to 620 ‘relevant’ publications that were obtained from online databases. This study references a total of 305 publications. The inclusion criteria for this review study encompassed the following aspects: The search was conducted on the entire text of the papers to ensure that no relevant articles were excluded due to the absence of the searched keywords in the abstracts or titles. The search was restricted to papers written in the English language. Every other language is not included. The publication period spanned from 1990 to 2024, focusing mostly on the last five years.

Out of the 450 papers that were finally reviewed, 145 were excluded due to their lower degree of relevance. The excluded papers were the outcome of the authors working autonomously to reduce bias and adhere to the eligibility criteria. The minor inconsistencies were resolved through consensus, considering the established selection criteria. The duration of this research spanned from March 2024 to August 2024. The process consisted of five stages: Preparation, formulation of research questions and queries, retrieval of data, analysis of data, synthesis of data, and presentation of results. The PRISMA 2020 statement was utilized to streamline the preparation and reporting of the present review. However, a Prisma diagram is omitted from the paper since this review is not a systematic review.

After extracting the data, we conducted analysis, assessed the quality, and synthesized the information. During the synthesis phase, a thorough analysis, primarily qualitative in nature, has been conducted on the information obtained from the reviewed articles, reports, and papers. This was essential for facilitating the classification and integration of data. The objective was to accurately outline the primary domains of investigation pertaining to our research inquiries. The results and discussion Section 2 and Section 3 contain significant findings and conclusions, which are supported by references to the included papers. This specific review has not undergone any statistical analysis. The synthesis of the identified studies, including different perspectives, gaps, and trends, is presented. The literature review has resulted in the proposal of best practice recommendations. Ultimately, this research highlights the results, effects, and significance it has on the scientific community. Additionally, this study also provides further recommendations for future research.

## Figures and Tables

**Table 1:** Symptoms associated with long COVID.

Long COVID Symptoms ^* CNS Symptoms in Bold *^	Indicative Studies
**Headache, Fever** **Fatigue** **Anosmia-Taste/smell loss**	[29,37,47,95,96]
Diarrhea Abdominal pain	[38,40,47]
**Cognitive Disfunction “Brain fog”** Peripheral Neuropathy **Peripheral neuropathy**	[30,31,32,36,40,41]
Arrythmia, Tachycardia Chest pain	[12,41,97,98]
Dyspnea Dry cough	[37,38,40,46,99]
**Insomnia** **Anxiety or depression** **Stress sensitivity** Hair loss **Mood disorders** **Psychiatric symptoms**	[12,22,31,32,33,42,49,52,100]

**Table 2:** Summary of key neurobeneficial effects of *Olea europaea* L. polyphenols.

Polyphenol	Study Type	Model/Cell Type	Effects *	Reference
**Hydroxytyrosol (HT)**	**In vitro**	BV2 microglia and primary microglia cells	AI: Dose-dependent decrease of pro-inflammatory mediators (modulation of M1/M2 polarization), TLR4 (NF-κB p65 and ERK signaling)	[75]
			NP: Complete inhibition of α-syn aggregation with HT-acetate	[215]
	**In vivo**	Mouse	AI: Dose-dependent decrease of pro-inflammatory mediators, microglia/astrocyte activation	[75]
		*C. elegans*	NP: 76.2% inhibition of α-syn aggregation with HT-acetate	[215]
**Oleuropein (OL)**	**In vitro**	Molecular dynamics trajectory analysis	NP: Stabilizes α-syn monomer and nontoxic aggregates	[113]
		(LPS)-treated monocyte/macrophages (THP-1) and endothelial cells (HUVECs), senescent HUVECs and Poly(I:C)-treated small airway epithelial cells (hSAECs)	AI: Decreased pro-inflammatory mediators (IL-1β, TNF-α, IL-8, ICAM, VCAM) and release of IL-6. In hSAECs, modulates the expression of SOD2, NF-kB, ACE2 and TMPRSS2	[127]
		CD4+ T cells from PBMCs of healthy controls and rheumatoid arthritis patients	AI: Dose-dependent increase in frequency of CD4+ CD25+ FoxP3 Tregs, IL-10 and TGF-β production	[199]
	**In vivo**	3 mo 5XFAD AD model	AI: Inhibition of NF-κB, NLRP3 inflammasomes and RAGE/HMGB1 pathways NP: Reduction of total Aβ brain levels and enhanced BBB integrity and function	[200]
		*C. elegans*	AO: Decreased oxidative stress involving DAF-16/FOXO and SKN-1/NRF2, and HSP-16.2 NP: Decreasesd Aβ and tau aggregation	[216]
		Rotenone PD model	NP: Increased CREB and phosphorylation of Akt and GSK-3β; reduction of mitochondrial dysfunction by activation of enzyme complexes and downregulation of the proapoptotic markers	[222]
	**Clinical**	Probable mild AD patients	NP: Neurocognitive parameters stabilized or improved	[136]
**Oleocanthal (OC)**	**In vitro**	Adipocytes	AI: Decreased TNF-α induced IL-1β, COX-2 Decreased TNF-α induced MCP-1, CXCL-10, M-CSF Decreased TNF-α induced miR-155-5p, miR-34a-5p and let-7c-5p Increased PPARγ Decreased TNF-α induced NF-κB activation AO: Decreased TNF-α induced NADPH oxidase, SOD, GPX	[206]
		Murine peritoneal macrophages	AI: Decreased LPS-induced MAPK pathway, inflammasome cascade signaling pathway, IL-1β, IL-6, IL-17, INF-γ, and TNF-α AO: Decreased LPS-induced ROS production	[213]
			AO: Demonstrated ROS scavenger capacity against HOCl and O_2_^●−^	[133]
	**In vivo**	5XFAD AD model (females)	AI: COX inhibition, suppressed C3AR1 activity (via STAT3) AO: Decreased Aβ plaques and tau phosphorylation	[66]
		5XFAD AD model	AI: Decrease NF-κB pathway and NLRP3, OC only decreased RAGE/HMBG1 pathway NP: Decreased Aβ levels	[66]
		TgSwDI AD model, (6 months)	AI: Inhibition of NACHT, LRR, and NLRP3 NP: Restored BBB function, reduced Aβ pathology induced autophagy through activation of AMPK/ ULK1 pathway	[132]
	**Clinical**	Obese and prediabetic individuals	AI: Decreased IFN-γ NP: Increased total antioxidant status, decreased lipid and organic peroxides	[154]

* Anti-inflammatory (AI), Antioxidant (AO) and Neuroprotective (NP).

## Data Availability

Not applicable.

## Abbreviations

Absorption, distribution, metabolism, and excretion (ADME), α-synuclein (α-syn), adrenocorticotropic hormone (ACTH), Alzheimer’s disease (AD), amyloid-β (Aβ), amyotrophic lateral sclerosis, ALS), angiotensin converting enzyme 2 (ACE2),Apolipoprotein E ϵ4 allele (APOE4), blood brain barrier (BBB), brain-derived neurotrophic factors (BDNFs), central nervous system (CNS), chronic fatigue syndrome (CFS), cyclooxygenase-1 and–2 (COX-1, COX-2), deep brain stimulation (DBS), extra virgin olive oil (EVOO), glial fibrillary acidic protein (GFAP), hydroxytyrosol (HT), intensive therapy unit (ITU); interferon-beta (IFNβ), interleukin-1 beta (IL-1β), mast cell activation syndrome (MCAS), Mediterranean Diet (MD), multiple sclerosis (MS), myalgic encephalomyelitis (ME), nuclear factor-kappa B (NF-κB); neurofibrillary tangles (NFTs), n-methyl-d-aspartate (NMDA), oleocanthal (OC), oleuropein (OL), Parkinson’s disease (PD), peripheral blood mononucleotides (PBMCs), post-acute sequelae of SARS-CoV-2 (PASC), receptor-binding domain (RBD), superoxide dismutase (SOD), tumor necrosis factor (TNF)-α, toll-like receptor 4 (TLR4).
